# Physiological Responses and Expression Profile of *NADPH Oxidase* in Rice (*Oryza Sativa*) Seedlings under Different Levels of Submergence

**DOI:** 10.1186/s12284-016-0074-9

**Published:** 2016-01-25

**Authors:** Yu-Sian Wu, Chin-Ying Yang

**Affiliations:** Department of Agronomy, National Chung Hsing University, Taichung, 40227 Taiwan

**Keywords:** Rice, Partial submergence, Full submergence, ROS, Antioxidant enzyme activity, Rboh, Hypoxia

## Abstract

**Background:**

Flooding due to global climate change is a serious problem that frequently decreases crop yields. Rice fields in flood-prone areas often experience full or partial submergence. Submergence has an adverse effect on internal oxygen availability, sugar status and survival. Complete submergence imposes severe pressure on plants, principally because the excess water in their surroundings deprives them of certain basic resources such as oxygen, carbon dioxide and light for photosynthesis. To better understand the mechanisms involved under different levels of flooding, it is necessary to further observe physiological responses and to identify the Rboh genes involved and determine how they are regulated during submergence.

**Results:**

In this study, significant physiological changes were observed in plant height, leaf sheath elongation and chlorophyll a, b and total content under partial and full submergence treatments. Senescence-regulating genes were severely affected under full submergence. Additionally, intracellular oxidative homeostasis was disrupted by overproduction of H_2_O_2_ and O_2_^−^, which affected cell viability and antioxidant enzyme activity, under different levels of submergence. Quantitative RT-PCR analyses revealed that complex regulation of Rboh genes is involved under different levels of submergence.

**Conclusion:**

Our results demonstrated that the effect of physiological and the transcript levels of OsRboh genes were presented different responses to different levels of submergence in rice seedlings. There have different mechanism in intracellular to response different levels of submergence. Finally we discuss effects of the regulation of OsRboh expression and ROS production which was important to maintain homeostasis to help rice seedlings face different levels of submergence.

**Electronic supplementary material:**

The online version of this article (doi:10.1186/s12284-016-0074-9) contains supplementary material, which is available to authorized users.

## Background

The effect of global climate change on crop yields is an urgent issue. Waterlogging is a serious problem that affects crop gas exchange in low-lying rain-fed areas. Approximately 30 % of the world’s rice (*Oryza sativa*) farmlands are at a low elevation and irrigated by rain (Bailey-Serres et al. 2010). Under limited oxygen availability, photosynthesis and respiration are restricted, leading to an energy crisis, toxic products from anaerobic respiration and the accumulation of reactive oxygen species (ROS) in plant cells (Licausi and Perata 2009; Pucciariello et al. 2012a﻿; Yang and Hong 2015).

Several studies have focused on the important role of ROS during hypoxia signaling under full submergence conditions (Baxter et al. 2014; Fukao et al. 2011; Liu et al. 2015; Yang and Hong 2015 ). Plasma membrane NADPH oxidases in plants were discovered through their sequence similarity to mammalian respiratory burst NADPH oxidase subunit gp91^phox^ and have been named respiratory burst oxidase homologs (Rbohs) (Sagi and Fluhr 2006). Rbohs participate in ROS production and are involved in the oxidative burst in response to pathogens (Lherminier et al. 2009; Sagi and Fluhr 2006). Some reports have indicated that Rboh proteins possess a resistance function, such as AtRboh D and AtRboh F, which may be involved in triggering cell death in inoculated plants (Pogany et al. 2009; Vellosillo et al. 2010). AtRboh D and AtRboh F are involved not only in the hypersensitive response but also the ABA- and ethylene-induced stomatal response and are associated with a very complicated interaction between the ABA-, ethylene-, JA- and SA-signaling pathways (Desikan et al. 2006; Maruta et al. 2011). Both ethylene and H_2_O_2_ were demonstrated promote aerenchyma formation in rice stems in a dose-dependent manner (Steffens et al. 2011). In a recent study, the NADPH oxidase Rboh D was found to be involved in primary hypoxia signaling and modulate down-stream gene expression (Yang and Hong 2015).

Floods are major constraints to crop production worldwide that affect oxygen availability in the environment and lead to serious physiological damage. Transient flash floods leading to partial or complete submergence can affect crop growth and yield (Kato et al. 2014). In recent years, many reports have indicated that complete submergence has an adverse effect on internal oxygen availability, sugar status and survival (Bailey-Serres and Voesenek 2010; Winkel et al. 2013). Complete submergence imposes severe pressure on plants, principally because excess water in their surroundings deprives them of certain basic resources such as oxygen, carbon dioxide and light for photosynthesis (Sakagami et al. 2013). Paddy field rice is generally tolerant to waterlogging and partial submergence compared with other economic crops such as maize and soybean. Lowland rice (Flood Resistant 13A; FR13A) can restrict its elongation growth, economizing its carbohydrate reserves to enable the development of new leaves upon desubmergence (Fukao et al. 2011).

So far, these results have mainly been obtained from complete submergence studies. To better understand the mechanisms involved under different levels of flooding, it is necessary to further identify the Rboh genes involved and to determine how they are regulated during submergence. In this study, we characterized the physiological responses and the transcript profiles of nine *Rbohs* under different levels of flooding treatments in rice (*Oryza sativa* L. *japonica*, Tai keng 9). Moreover, we examined the accumulation of H_2_O_2_ and O_2_^−^ using DAB and NBT staining and the activities of antioxidant enzymes. This study provides evidence of differential regulation of OsRboh expression and ROS accumulation under partial or full submergence in rice.

## Results

### Partial Submergence Leads to Faster Plant Height Increases and Leaf Sheath Elongation at a Relatively Early Stage

**Fig. 1:**
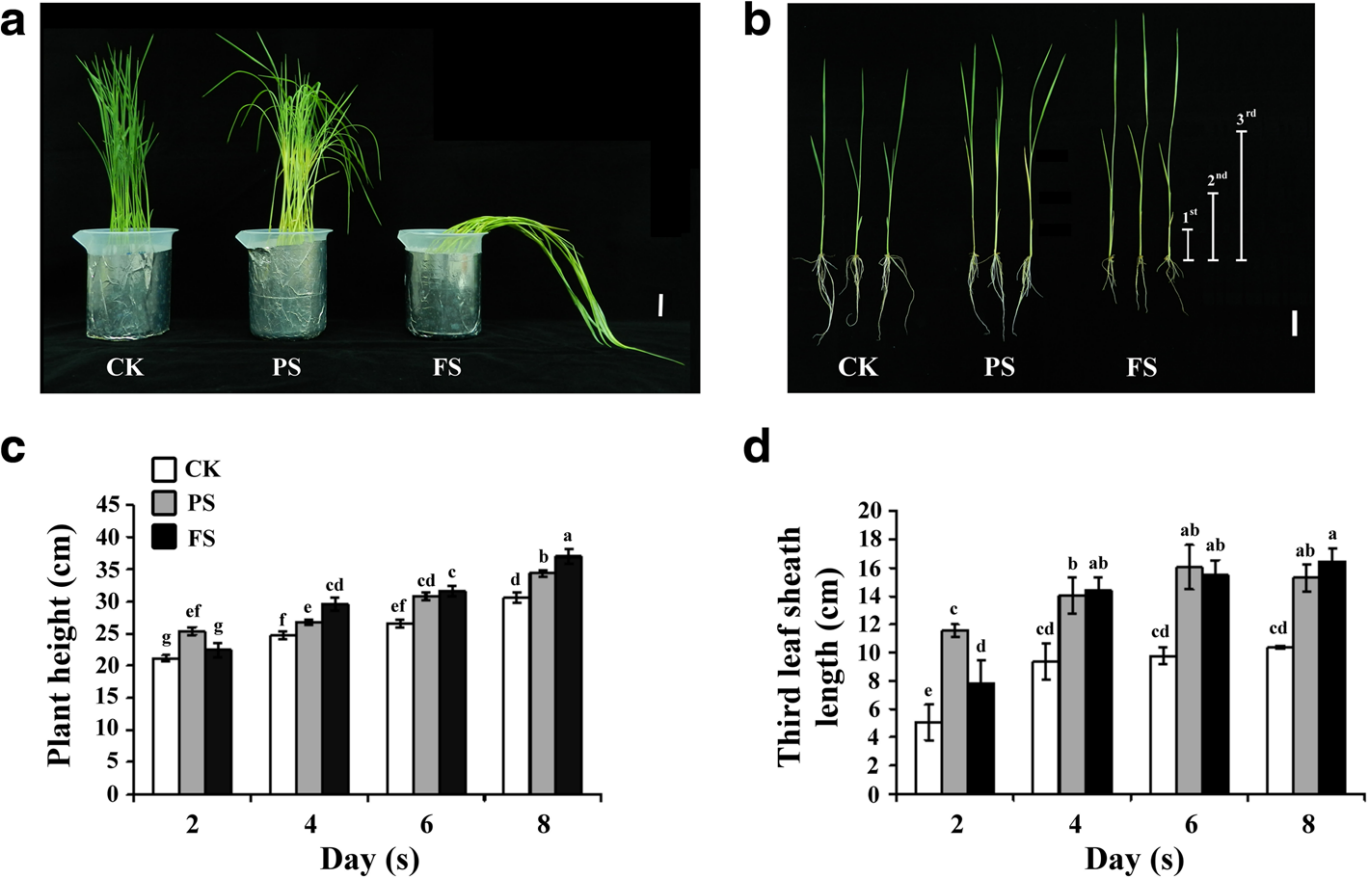
Characterization of rice (*Oryza sativa*) seedlings growth under different levels of submergence. **a** Photographs of 14 days-old rice seedlings after exposed different levels of submergence for 8 days. **b** The phenotypes of plant height after exposed different levels of submergence treatment for 8 days. (1st, 2nd and 3rd; length of the first, second and third leaf sheath). **c** Plant height of 14 days-old rice seedlings after treated submergence for 2, 4, 6 and 8 days. **d** The third leaves sheath length of 14 days-old rice seedlings after treated submergence for 2, 4, 6 and 8 days. Control check (CK), partial submergence (PS) and full submergence (FS). Bar = 3 cm. The data represent average values ± SD from 30 seedlings of each treatment obtained from six biologically independent experiments. Values with the different letters are significantly different at *P* < 0.05, according to post-hoc LSD test

To assess several physiological mechanisms associated with partial and full submergence in rice, plant height and leaf sheath length were determined in 14 days-old rice seedlings under partial submergence (PS) or full submergence (FS) for 2, 4, 6 and 8 days. The leaves of the rice seedlings were yellowish, thin and long, and could not remain upright under FS for 8 days compared with regular growth (control check; CK) and PS conditions (Fig. 1a and b). After PS treatment, the yellowish color was only present in tissue that had been submerged (Fig. 1a and b). During the partial submergence treatment period, plant height and the length of the 3^rd^ sheath were significantly increased after 2 days treatment. Subsequently, plant height and the length of the 3^rd^ leaf sheath increased in both PS and FS conditions compared with the control (Fig. 1c and d). There were no significant differences in the lengths of the 1^st^ and 2^nd^ leaf sheaths after PS or FS treatment compared with the control (Additional file 1: Figure S1). These results indicated that the plant height increase of rice seedlings during submergence was mainly affected by elongation of the 3^rd^ leaf sheath. Notably, partial submergence led to faster elongation at a relatively early stage compared with full submergence.

### The Chlorophyll Content and Senescence-regulating Genes are Severely Affected under Full Submergence

The photosynthetic apparatus and particularly PS II in rice seedlings is affected by submergence, which causes ROS damage to the photosystem and a decreased photosynthetic rate and chlorophyll content (Panda et al. 2008). The submergence tolerance regulator, SUBMERGENCE1A (SUB1A), is involved in acclimation responses during leaf senescence caused by prolonged darkness in rice (Fukao et al. 2012).

**Fig. 2:**
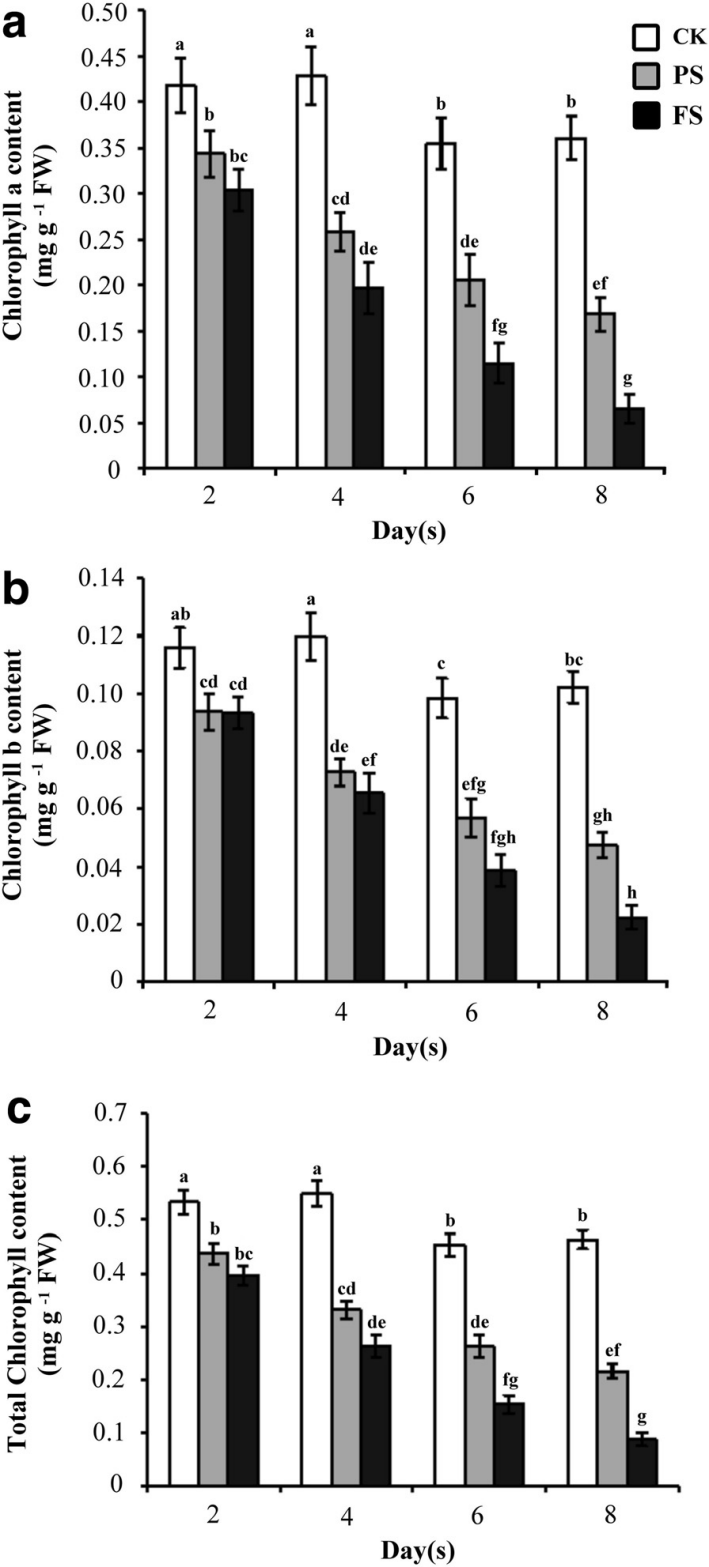
Different levels of submergence influence the content of chlorophyll in rice seedlings. The chlorophyll content of 14-days old seedlings after treated control (CK), partial submergence (PS) and full submergence (FS) conditions for 2, 4, 6 and 8 days. The content of chlorophyll a (**a**), chlorophyll b (**b**) and total chlorophyll (**c**) were determined. Values represent means ± standard deviation from six biologically independent experiments (*n* = 3). Values with the different letters are significantly different at *P* < 0.05, according to post-hoc LSD test

**Fig. 3:**
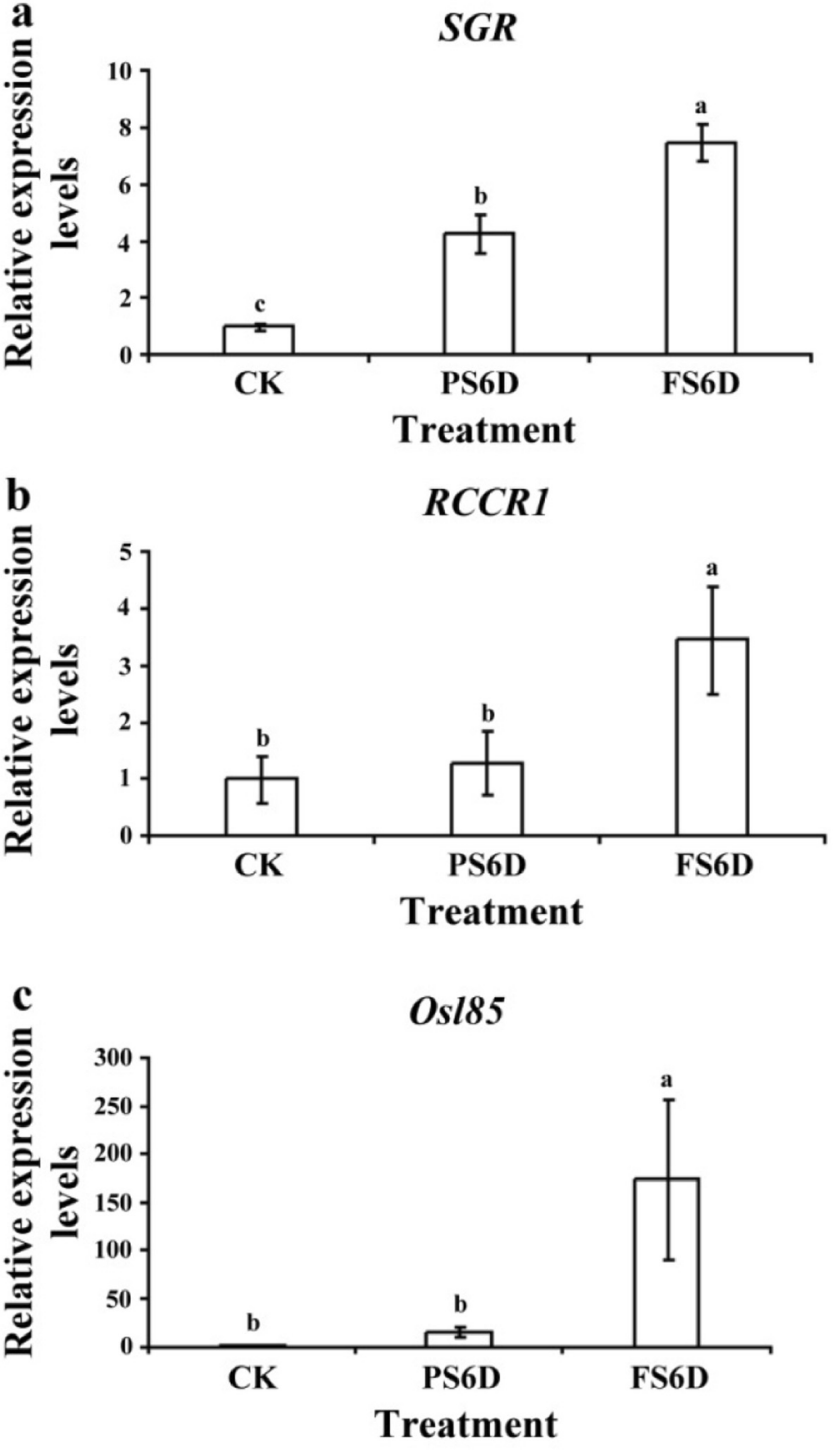
Transcript levels of gene associated with leaf senescence. Quantitative RT-PCR was used to quantify transcript levels of *Stay-Green* (*SGR*), *Red Chlorophyll Catabolite Reductase* (*RCCR*) and *Isocitrate Lyase* (*Osl85*) genes in 14 days-old rice seedlings after treated control (CK), partial submergence (PS) and full submergence (FS) conditions for 6 days. Relative amounts of transcripts were calculated and normalized to that of Ubiquitin mRNA. Values represent means ± standard deviation from five biologically independent experiment. Values with the different letters are significantly different at *P* < 0.05, according to post-hoc LSD test

To dissect the influence of chlorophyll content and the expression of senescence-regulating genes under different levels of submergence, we determined the chlorophyll a, b and total content after PS or FS treatment for 2, 4, 6 and 8 d in 14 days-old rice seedlings. The results showed that the chlorophyll a, b and total contents were significantly decreased with submergence treatment, particularly in the FS treatment (Fig. 2). To evaluate whether senescence-associated gene (SAG) expression was affected by different levels of submergence, we used quantitative RT-PCR to determine the mRNA expression of *Stay*-*Green* (*SGR*), which is necessary for chlorophyll degradation in light-harvesting complex II (Hortensteiner 2009), *Red Chlorophyll Catabolite Reductase* (*RCCR*), which functions in chlorophyll degradation in chloroplasts (Pruzinska et al. 2007), and *Osl85*, which encodes isocitrate lyase, a marker gene that is upregulated during dark-induced and natural leaf senescence (Yamada et al. 2014). The quantitative RT-PCR results revealed that the transcripts of these senescence-regulating genes were highly induced by FS treatment compared with PS treatment (Fig. 3). These results demonstrated that FS conditions severely affect the chlorophyll content, which is accompanied by senescence induction, in rice seedlings more than PS conditions.

### Full Submergence Leads to H_2_O_2_ and O_2_^−^ Overproduction that Disrupts Intracellular Oxidative Homeostasis

**Fig. 4:**
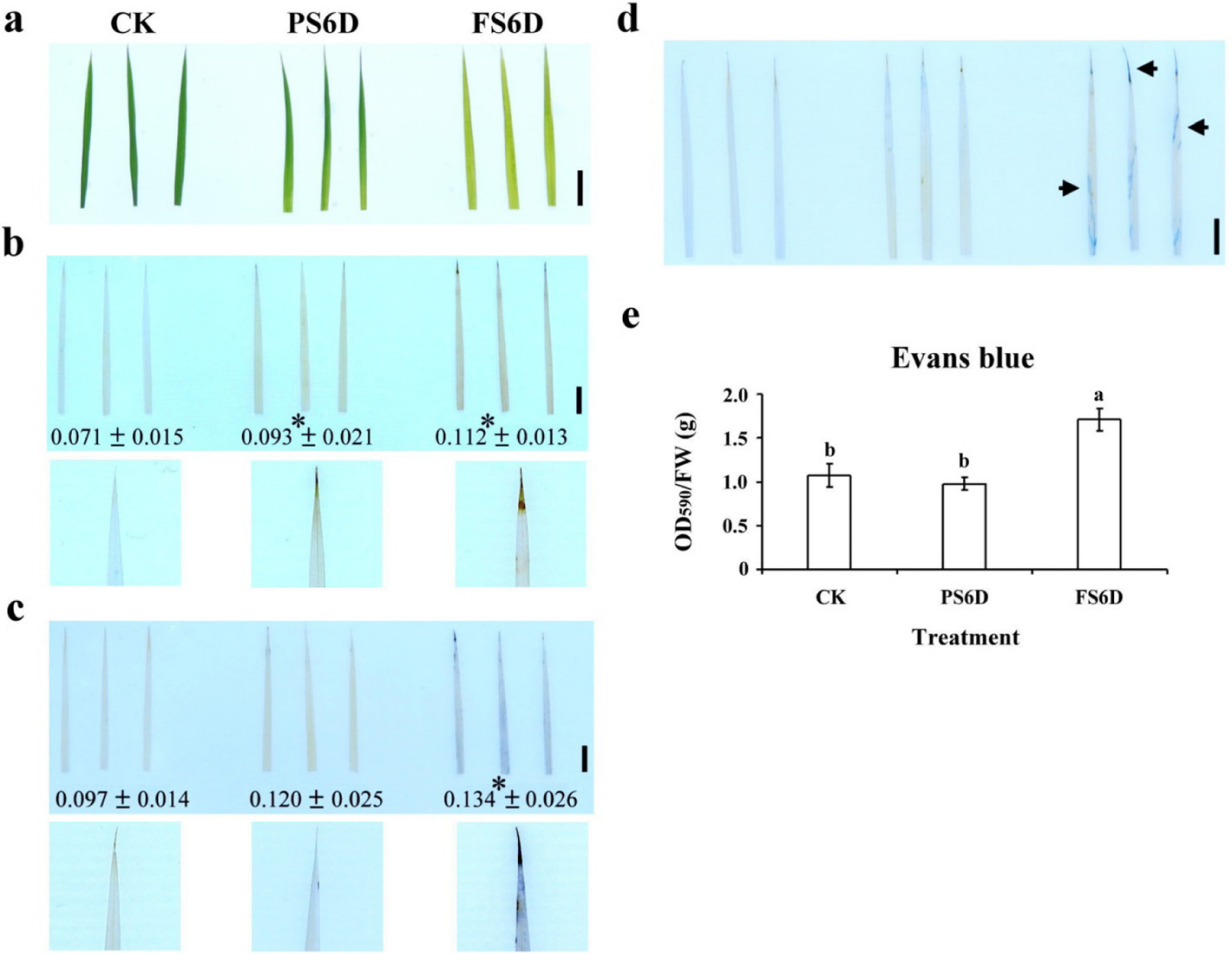
DAB, NBT and Evans blue staining for the detection of H_2_O_2_, O_2_^−^ and cell death in rice seedlings under different levels of submergence. **a** Phenotypes of 14 days-old rice seedlings after treated control (CK), partial submergence (PS) and full submergence (FS) for 6 days. **b** and **c**, DAB and NBT staining of H_2_O_2_ and O_2_^−^ in detached leaves of 14 days-old rice seedlings after treated control (CK), partial submergence (PS) and full submergence (FS) for 6 days. Same results were obtained in three independent experiments. Photograph shows results of three independent leaves. **d** and **e**, Evans blue staining and cell death analysis (see materials and methods for details). Bar = 1 cm. The data represent average values ± SD from five biologically independent experiments. Values with the different letters are significantly different at *P* < 0.05, according to post-hoc LSD test

Several studies have shown that ROS are common components of biochemical changes in chloroplasts, mitochondria and peroxisomes when plants are subjected to oxygen deprivation conditions (Blokhina et al. 2003; Damanik et al. 2010). To investigate ROS accumulation, H_2_O_2_ and O_2_^−^ accumulation under different levels of submergence was evaluated by DAB and NBT staining. The 3^rd^ leaves in the submerged area appeared yellow after PS or FS treatment for 6 days (Fig. 4). As shown in Fig. 4b and c, H_2_O_2_ and O_2_^−^ were accumulated to higher levels in FS than in PS conditions, especially in the tips of leaves. We also determined the cell viability by Evans blue staining, and the results showed significant cell death in the tips and blades of leaves in FS conditions (Fig. 4d and e).

**Fig. 5:**
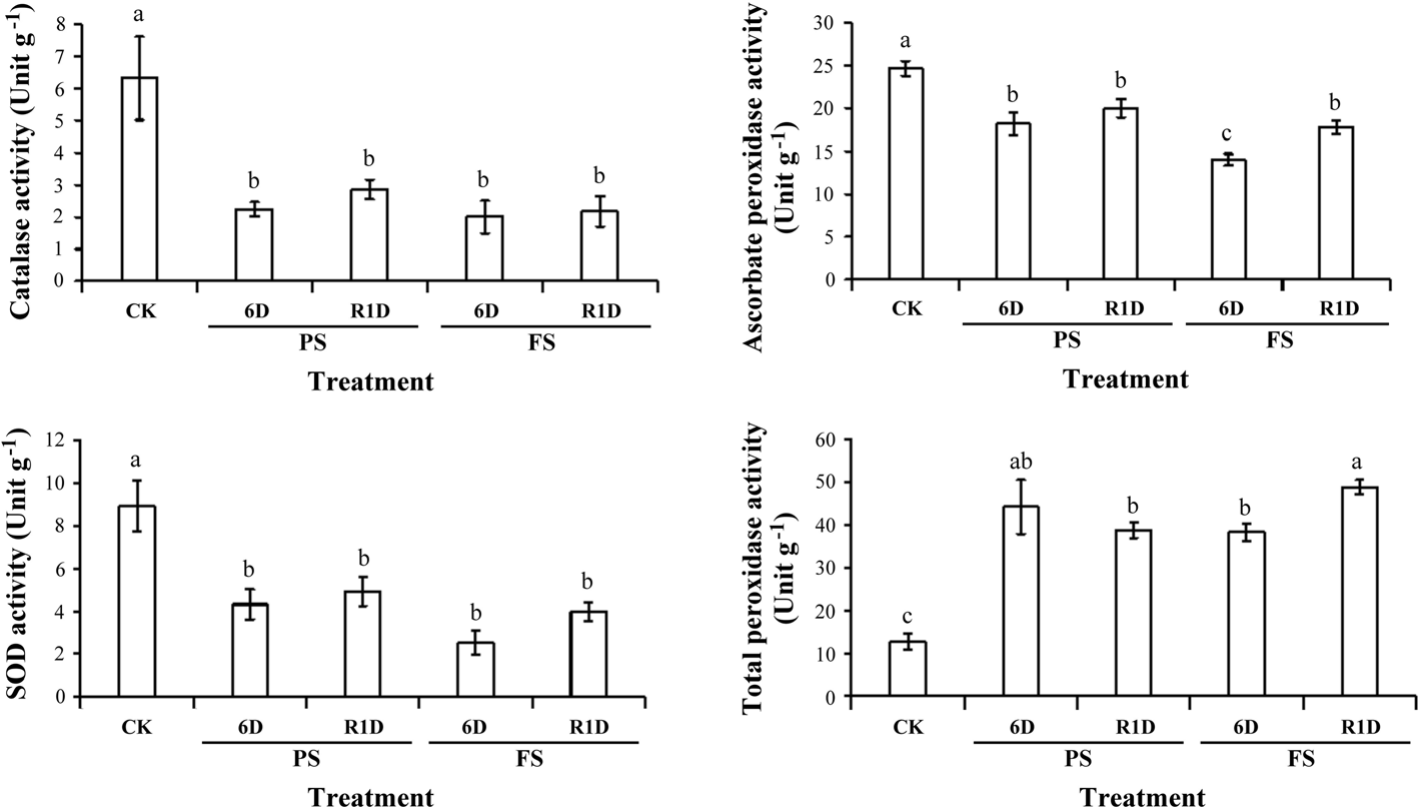
The activities of CAT, APX, SOD and POX in rice seedlings under different levels of submergence. Enzyme activity was detected from detached shoots of 14 days-old rice seedlings after treated control (CK), partial submergence (PS) and full submergence (FS) for 6 days then recovery 1 day (R1D). The catalase (CAT), ascorbate peroxidase (APX), superoxide dismutase (SOD) and total peroxidase (POX) activity were determined, respectively. The data represent average values ± SD from six biologically independent experiments. Values with the different letters are significantly different at *P* < 0.05, according to post-hoc LSD test

To investigate the cellular scavenging capacity of the antioxidative enzymes in rice seedlings under different levels of submergence, the activities of antioxidant enzymes such as catalase (CAT), ascorbate peroxidase (APX), superoxide dismutase (SOD) and total peroxidase (POX) were evaluated. The CAT, SOD and APX activities were decreased under PS and FS treatment for 6 days and recovery for 1 day compared with the control plants. The APX activity was decreased more under FS treatment for 6 days than PS treatment. The POX activity was significantly increased under PS and FS treatment for 6 days and recovery for 1 day (Fig. 5). Thus, our results suggest that the overproduction of H_2_O_2_ and O_2_^−^ disrupts intracellular oxidative homeostasis, which affects cell viability and antioxidant enzyme activity under different levels of submergence.

### Transcript Profiles of *OsRboh* Genes under Different Levels of Submergence

**Fig. 6:**
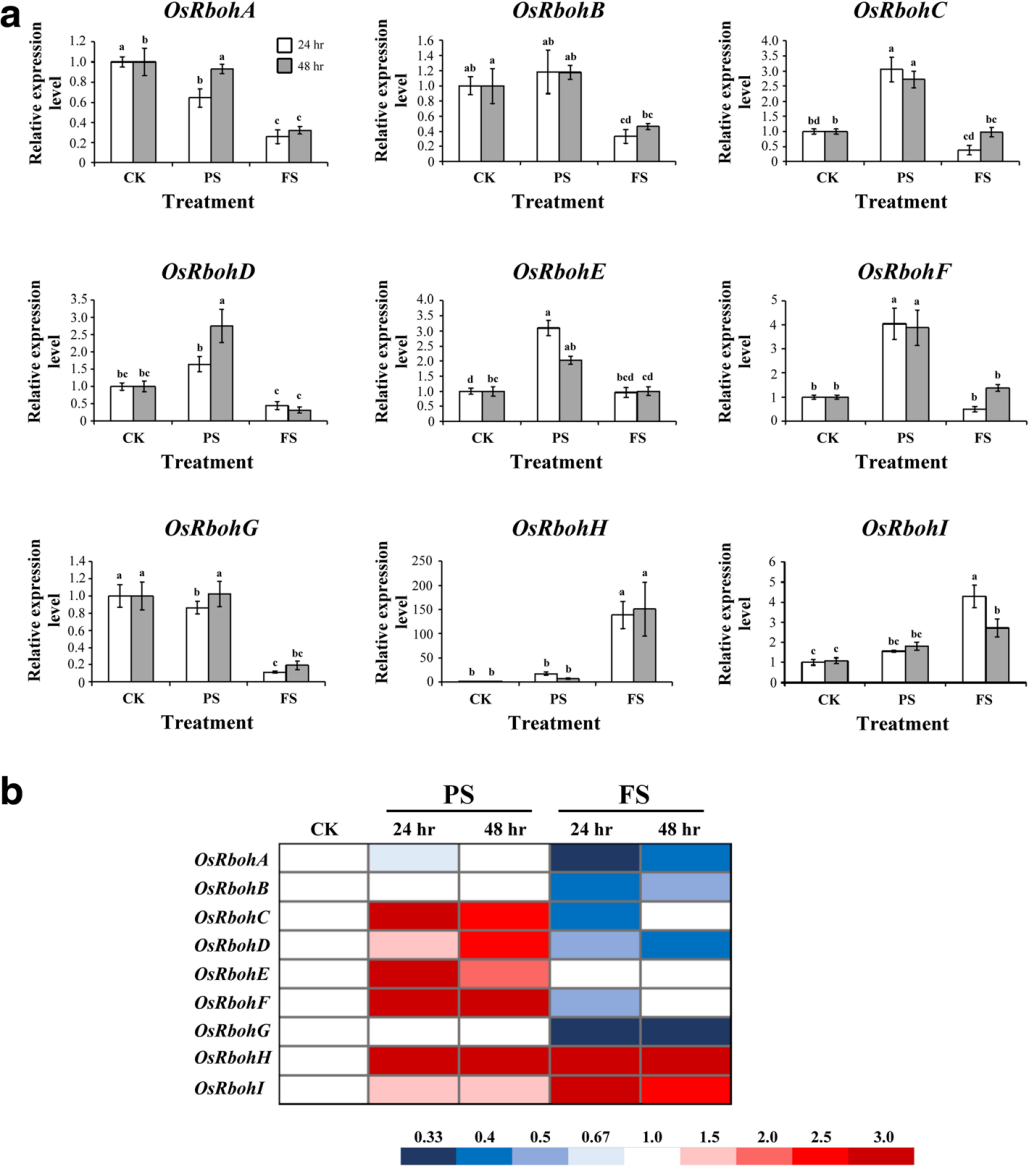
Transcript profiles of *Rboh* genes in rice seedlings under different levels of submergence. **a** quantitative RT-PCR analyses of transcript levels of *Rbohs* genes in 14 days-old rice seedlings after treated control (CK), partial submergence (PS) and full submergence (FS) conditions. **b** fold change were presented by different color. Total RNAs were isolated from shoots of 14-days-old seedlings after 24 and 48 h submergence treatment and levels of *Rboh A*–*I* mRNA were determined. Relative amounts of transcripts were calculated and normalized to that of *ubiquitin* mRNA. Values represent means standard deviation from five biologically independent experiments. Values with the different letters are significantly different at *P* < 0.05, according to post-hoc LSD test

H_2_O_2_participates in hypoxia signal transduction to modulate the expression of a set of genes encoding heat-shock proteins and other groups of ROS-mediated proteins (Pucciariello et al. 2012b). NADPH oxidase enzymes are key players in H_2_O_2_ production. Previously, we showed that *AtRboh* genes are involved in hypoxia signaling, and revealed the different transcript profiles of *Rboh* genes under hypoxic stress in *Arabidopsis*. AtRboh D plays a major role at an early stage to modulate the expression of down-stream hypoxia-inducible genes under hypoxic stress (Yang and Hong 2015). Phylogenetic analysis of the corresponding corrected protein sequences of ten *Arabidopsis* and nine rice Rboh proteins were obtained from the TAIR (http://www.arabidopsis.org/) and Phytozome (https://phytozome.jgi.doe.gov/) databases (Additional file 2: Figure S2). To characterize the expression profiles of the nine Rboh genes in rice seedlings during submergence, their transcript levels were determined by quantitative RT-PCR under different levels of submergence. The transcript levels of the *Rboh C* (*Os05g45210*), *D* (*Os05g38980*), *E* (*Os01g61880*) and *F* (*Os08g35210*) genes increased under PS conditions, the transcript levels of *Rboh H* (*Os12g35610*) and *I* (*Os11g33120*) genes increased under FS conditions, the transcript levels of *Rboh A* (*Os01g53294*) decreased under PS treatment for 24 h and FS treatment for 24 or 48 h, the transcript levels of the *Rboh B* (*Os01g25820*) and *G* (*Os09g26660*) genes decreased under FS conditions (Fig. 6a and b). These results not only demonstrate that Rboh genes are involved in different levels of submergence, but also reveal the complex regulation of Rboh genes in hypoxia signaling in rice seedlings.

## Discussion

Rice fields in flood-prone areas often experience full or partial submergence. The many visible symptoms of injury caused by full or partial submergence include an initial phase of fast elongation by one or more leaves accompanied by yellowing of old leaves, and slow growth in the dry mass of roots and shoots (Jackson and Ram 2003; Kato et al. 2014; Sarkar and Bhattacharjee 2012). The survival strategies of flood-tolerant plants are characterized as low-O_2_ escape syndrome (LOES) and low-O_2_ quiescence syndrome (LOQS) (Voesenek and Bailey-Serres 2015). LOES facilitates escape from submergence stress and includes upward growth of leaves (hyponastic growth) and petiole/stem elongation initiated by ethylene accumulation (Jackson 2008). To further dissect the effects of different levels of flooding, we investigated the physiological and molecular responses of rice seedlings under partial and full submergence. In this study, our physiological experiment demonstrated that the plant height of rice seedlings was increased, which was mainly affected by elongation of the 3^rd^ leaf sheath, during submergence. Furthermore, partial submergence led to faster elongation at a relatively early stage compared with full submergence (Fig. 1). Therefore, an early physiological response to partial submergence is the elongation of leaf sheaths, which implies sensing of different levels of submergence and different regulation mechanisms in rice seedlings.

A significant reduction of Rubisco activity, inhibition of the CO_2_ photosynthetic rate and ROS accumulation occurs when plants are exposed to flooding conditions for prolonged periods (Kamal and Komatsu 2015; Panda et al. 2008; Yang and Hong 2015; Yang 2014). The catabolism of chlorophyll is regulated by natural leaf senescence and environmental stress (Lim et al. 2007). Our results showed that the chlorophyll a, b and total contents were significant decreased by submergence treatment, particularly FS treatment (Fig. 2). The transcript accumulation of genes associated with senescence was highly induced by FS treatment compared with PS treatment (Fig. 3). These results reveal that different levels of submergence not only affect SAG expression, but also lead to a reduction of chlorophyll content. Notably, a characteristic enzyme gene of the glyoxylate cycle, *Osl85* (*isocitrate lyase*), was significantly induced after FS treatment. This seems to indicate that the glyoxylate cycle is involved in the response of rice seedlings to different levels of submergence.

Under submergence, the O_2_ concentration of water is lower than that of air, leading to suppressed respiration in roots and enhanced fermentation (Nishiuchi et al. 2012; Yin et al. 2013). This metabolic shift can cause toxicity from ethanol accumulation. The most common group of toxic intermediates produced is ROS. We demonstrated that H_2_O_2_ and O_2_^−^ accumulation was highest in the tips of leaves by histochemical staining and observed significant cell death in FS conditions (Fig. 4). Antioxidative enzyme activity detection showed that POX activity was significantly increased under PS and FS treatment for 6 days and recovery for 1 day (Fig. 5). Thus, the overproduction of H_2_O_2_ and O_2_^−^ disrupts intracellular oxidative homeostasis, which affects cell viability and antioxidant enzyme activity under different levels of submergence.

Previously, we characterized the transcription profiles of ten *Rbohs* in *Arabidopsis* under hypoxic stress and found Rboh D is involved in primary hypoxia signaling to modulate the expression of hypoxia-inducible genes under hypoxic stress (Yang and Hong 2015). Here, we showed that the expression profiles of nine Rboh genes in rice seedlings differed under different levels of submergence (Fig. 6), indicating Rboh genes are involved in responses to different levels of submergence. As in *Arabidopsis*, our data reveal the complex regulation of Rboh genes in hypoxia signaling in rice seedlings.

## Conclusions

Although flooding leads to chlorophyll content decreased and accompanied senescence induction, the transcript levels of OsRboh genes was presented different responses to different levels of submergence in rice seedlings. Our results demonstrate that have different mechanism in intracellular to response different levels of submergence. The regulation of OsRboh expression and ROS production was important to help rice seedlings face different levels of submergence.

## Methods

### Plant Materials, Growth Conditions and Stress Treatment

Rice (*Oryza sativa, japonica.*) Tai-keng 9 (TK9) was used in this study. Seeds were surface sterilized via immersion in a 3 % sodium hypochlorite solution for 30 min, and were then thoroughly rinsed with distilled water. Seeds were subsequently placed on wet filter paper for 3 day at 28 °C in a 16-h-light (236 μmolm^−2^s^−1^)/8-h-dark cycle in a growth chamber. The germinated seeds were transplanted onto iron grid in the beaker with Kimura B solution. For submergence treatment, 14-day old seedlings (2–3 leaf stage) were transferred into water box (W:L:H, 40 cm × 40 cm × 60 cm) filled with 23 cm high of water for partial submergence (PS) which the water level reached half of plants height or filled with 50 cm high of water for full submergence (FS). After each treatment, sampled tissue was immediately frozen in liquid nitrogen and stored in −80 °C until use.

### Measurement of Plant Height, Length of Leaf Sheath and Chlorophyll Content

After 14-day old seedlings were treatments by PS or FS for 2, 4, 6 and 8 days, the length of each shoot and leaf sheath (at least 30 plants each) were recorded for indicated times. Experiments were repeated five times independently. For chlorophyll content assays, chlorophyll a, b and total contents were extracted from 50 mg of shoot tissue in 2 mL of sodium phosphate buffer (50 mM pH 6.8). Add 40 μL extracted solution in 960 μL 99 % ethanol then incubated for 30 min at RT in the dark with gentle shaking. After centrifugation at 4 °C for 15 min at 1000  *g*, the absorbance of the supernatant was measured at 665 and 649 nm with a spectrophotometer (Metertec SP8001).

### Assay of O_2_^−^ and H_2_O_2_ Accumulation and Cell Viability by Histochemical Staining Method

Accumulation of O_2_^−^ in cell was visualized by the nitroblue tetrazolium (NBT) staining method. 14-day old seedlings after submergence treatment for 6 days, the 3 ^rd^ leaves were immersed into 1 mM NBT solution prepared in 10 mM phosphate buffer (pH 7.8) at RT under dark for 8 h. When blue spots appeared, the pigments were removed from leaves by boiling in 75 % ethanol for 5 min. Accumulation of H_2_O_2_ in cells was visualized by the 3, 3′ -diaminobenzidine (DAB) staining method as described in detail previously (Yang and Hong 2015). In the stained leaves, H_2_O_2_ is visualized as reddish-brown coloration. The leaves were photographed with a digital camera and the intensity of the colour in the NBT- or DAB-stained area was measured using Photoshop image software. Experiments were repeated three times independently. The stained intensity was measured at least 10 leaves for each biological repeat. Cell viability assay was visualized by the Evans blue staining. 14-day old seedlings after submergence treatment for 6 days, the 3 ^rd^ leaves were collected, weighed and immersed into 0.2 % Evans blue solution for 3 h with gentle shaking. The de-stained leaves (0.05 g/each) were homogenized with liquid nitrogen in 500 μL of 10 % SDS followed by the addition of 500 μL of de-ionized water. The solutions were centrifuged at 15,800 *g* for 30 min at 4 °C, the absorbance of the supernatant was measured at 590 nm with a spectrophotometer (Metertec SP8001). Experiments were repeated six times independently.

### Determination of Antioxidative Enzyme Activity

The shoot samples (50 mg) were excised and immediately used for enzyme extraction. The shoot tissues were homogenized with 50 mM sodium phosphate buffer (pH 6.8) with liquid nitrogen. The homogenate was centrifuged at 12,000 *g* for 20 min and the resulted supernatant was used for determination of enzyme activity. For CAT activity was performed as described previously (Chao et al. 2012; Kato and Shimizu 1985). The decrease in H_2_O_2_ was determined as the decrease in absorbance at 240 nm with a spectrophotometer (Metertec SP8001). The activity was calculated by the extinction coefficient (40 mM^−1^ cm^−1^ at 240 nm) for H_2_O_2_. One unit of CAT was defined as the amount of enzyme that degraded 1 μmol H_2_O_2_ per min. For SOD activity was determined according to Chao et al. (2012). The reaction mixture contained 100 mM triethanolamine- diethanolamine buffer (pH 7.4), EDTA/MnCl_2_ (100 mM/50 mM, pH 7.4), 7.5 mM β-NADH, 10 mM 2-mercaptoethanol, and enzyme extract. The reaction was started by the addition of β-NADH and absorbance was measured at 340 nm for 1 min. One unit of SOD was defined as the amount of enzyme that inhibited by 50 % the rate of β-NADH oxidation. For APX activity, the decrease in ascorbic acid (AsA) concentration was determined as the decline in absorbance at 290 nm and activity was calculated by the extinction coefficient (2.8 mM^−1^ cm^−1^ at 290 nm) for AsA (Chao et al. 2012; Nakano and Asada 1981). For total peroxidase activity was performed as described previously (Lin and Kao 1999; MacAdam et al. 1992). The shoot tissues were homogenized with 50 mM potassium phosphate buffer (pH 5.8) containing 0.8 M KCl with liquid nitrogen to extract both soluble and ionically bound (‘total’) peroxidase. The reaction mixture contained 50 mM potassium phosphate buffer (pH 5.8), 21.6 mM guaiacol, 39 mM H_2_O_2_ and enzyme extract. The reaction was started by the addition of H_2_O_2_ and absorbance was measured at 470 nm for 1 min. The activity was calculated by the extinction coefficient (26.6 mM^−1^ cm^−1^ at 470 nm) for tetraguaiacol. One unit of peroxidase was defined as the amount of enzyme that caused the formation 1 μmol tetraguaiacol per min.

### Quantitative RT-PCR Analyses

Shoot samples were collected from seedlings and frozen until analysis. RNA was isolated from frozen tissues by TRIzol reagent (Invitrogen, Carlsbad, CA, USA). Total RNA samples from shoots were first treated with DNase I and then reverse transcribed into cDNA by Moloney murine leukemia virus reverse transcriptase (Invitrogen). Quantitative RT-PCR was performed using an Illumina Eco Real-Time PCR system (Biogenesis Technology, San Diego, USA) with Power SYBR Green PCR Master Mix (Applied Biosystems), according to the manufacturer’s instructions. The relative expression level of each gene was quantified using the comparative threshold cycle method, as described in the manufacturer’s instructions for the Eco™ Real-Time PCR System User Guide. The *ubiquitin* (*Os03g13170*) gene was used as an internal control to normalize the cDNA levels. Data were analyzed using the EcoStudy software, version 4.0 (Biogenesis Technology). Amplification conditions were as follows: 94 °C for 5 min, and then 45 cycles at 94 °C for 15 s, 55 °C for 15 s and 72 °C for 30 s. The primers used for quantitative RT-PCR analyses were listed in Table 1. Quantitative RT-PCR experiments were repeated least six times independently in duplicate, and the data were averaged.

**Table 1:** Primers used for quantitative RT-PCR experiments

Gene name	NCBI accession	Forward primer sequence	Reverse primer sequence	Product size (bp)
*ubiquitin*	Os03g13170	5′-aaccagctgaggcccaaga-3′	5′-acgattgatttaaccagtccatga-3′	77
*SGR*	Os09g36200	5′-cgcatgcaatgtcgccaaatg-3′	5′-gctcaccacactcattccctaaag-3′	138
*RCCR1*	Os10g25030	5′-gcaccttctcactgacagcaatac-3′	5′-accacgcactatctcttccaagg-3′	83
*Osl85*	Os07g34520	5′-catgggcaaaggagttactgaagag-3′	5′-ggatttggcaagaacatggctg-3′	88
*OsRbohA*	Os01g53294	5′-gtcttatgcagtcatgaatgtaca-3′	5′-gaataatatacagttaattagcct-3′	129
*OsRbohB*	Os01g25820	5′-cctagtggaagaagctgtgct-3′	5′-cactatgaaagggaacatcacaa-3′	119
*OsRbohC*	Os05g45210	5′-tgttttagggatggttttacac-3′	5′-tgtacagacagaaggttaacgt-3′	94
*OsRbohD*	Os05g38980	5′-gaccagaccaggaaaaaaacaccaa-3′	5′-acacagaaagagttgctaaccgatg-3′	85
*OsRbohE*	Os01g61880	5′-catcgtgcatagattctgga-3′	5′-catgcattcccactgttcca-3′	62
*OsRbohF*	Os08g35210	5′-tcgtctatcatagatatacatg-3′	5′-cgtgtactttggtgacctcag-3′	85
*OsRbohG*	Os09g26660	5′-aagcgttgctaattttcgctat-3′	5′-gagaggatgtttttttgaacgg-3′	117
*OsRbohH*	Os12g35610	5′-gtacaattatatacagattaatg -3′	5′-cgaacaaccaatcactcactaa-3′	67
*OsRbohI*	Os11g33120	5′-tggccagataatttcatcggtt-3′	5′-gctactctaagtattacaaagta-3′	101

## Additional files

Additional file 1: Figure S1. Characterization of rice (*Oryza sativa*) seedlings growth under different levels of submergence. The first (a) and second (b) leaves sheath length of 14 days-old rice seedlings after treated submergence for 2, 4, 6 and 8 days. Control check (CK), partial submergence (PS) and full submergence (FS). The data represent average values ± SD from 30 seedlings of each treatment obtained from six biologically independent experiments. Values with the different letters are significantly different at *P* < 0.05, according to post-hoc LSD test. (JPG 1543 kb) Additional file 2: Figure S2. Phylogenetic tree of members of Rbohs protein family. Phylogenetic tree of 10 *Arabidopsis* Rboh proteins and 9 rice (Oryza sativa) related Rboh proteins. The phylogenetic tree was constructed by the Neighbor Joining algorithm (Saitou and Nei 1987) implemented in the MEGA 6 software package (Tamura et al. 2013). (JPG 614 kb)

## Abbreviations

APX: ascorbate peroxidase
CAT: catalase
FS: full submergence
H_2_O_2_: hydrogen peroxide
POX: total peroxidase
PS: partial submergence
Rbohs: respiratory burst oxidase homologs
ROS: reactive oxygen species
RT-PCR: reverse transcription-polymerase chain reaction
SOD: superoxide dismutase
