# Supplementary figures and images for: Physiological Responses and Expression Profile of NADPH Oxidase in Rice (Oryza Sativa) Seedlings under Different Levels of Submergence
Source: Rice (N Y). 2016 Jan 25;9:2. doi: 10.1186/s12284-016-0074-9 (PMC4726645; doi:10.1186/s12284-016-0074-9)

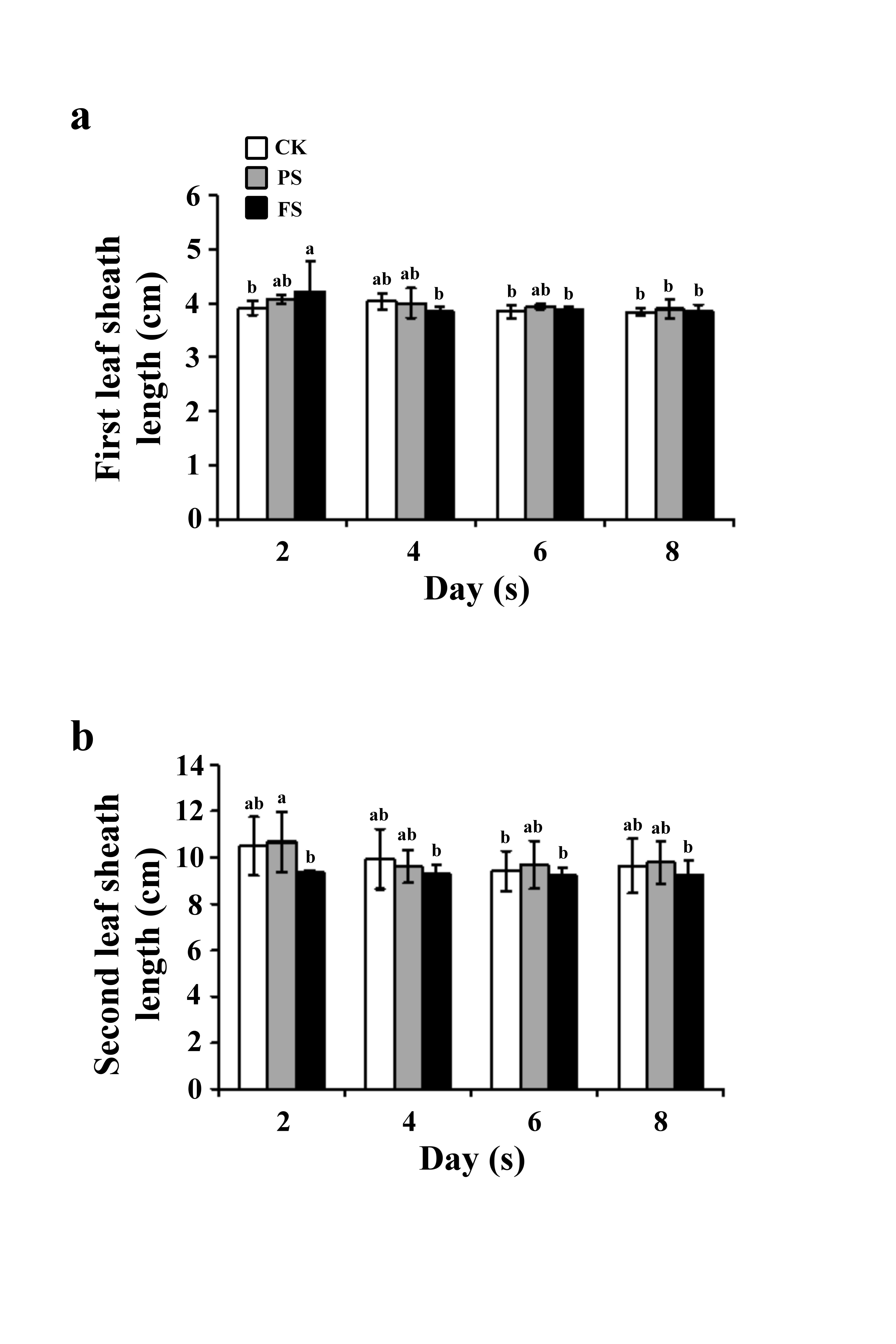

Supplement: Additional file 1: Figure S1. — Characterization of rice (Oryza sativa) seedlings growth under different levels of submergence. The first (a) and second (b) leaves sheath length of 14 days-old rice seedlings after treated submergence for 2, 4, 6 and 8 days. Control check (CK), partial submergence (PS) and full submergence (FS). The data represent average values ± SD from 30 seedlings of each treatment obtained from six biologically independent experiments. Values with the different letters are significantly different at P < 0.05, according to post-hoc LSD test. (JPG 1543 kb) [file 12284_2016_74_MOESM1_ESM.jpg]

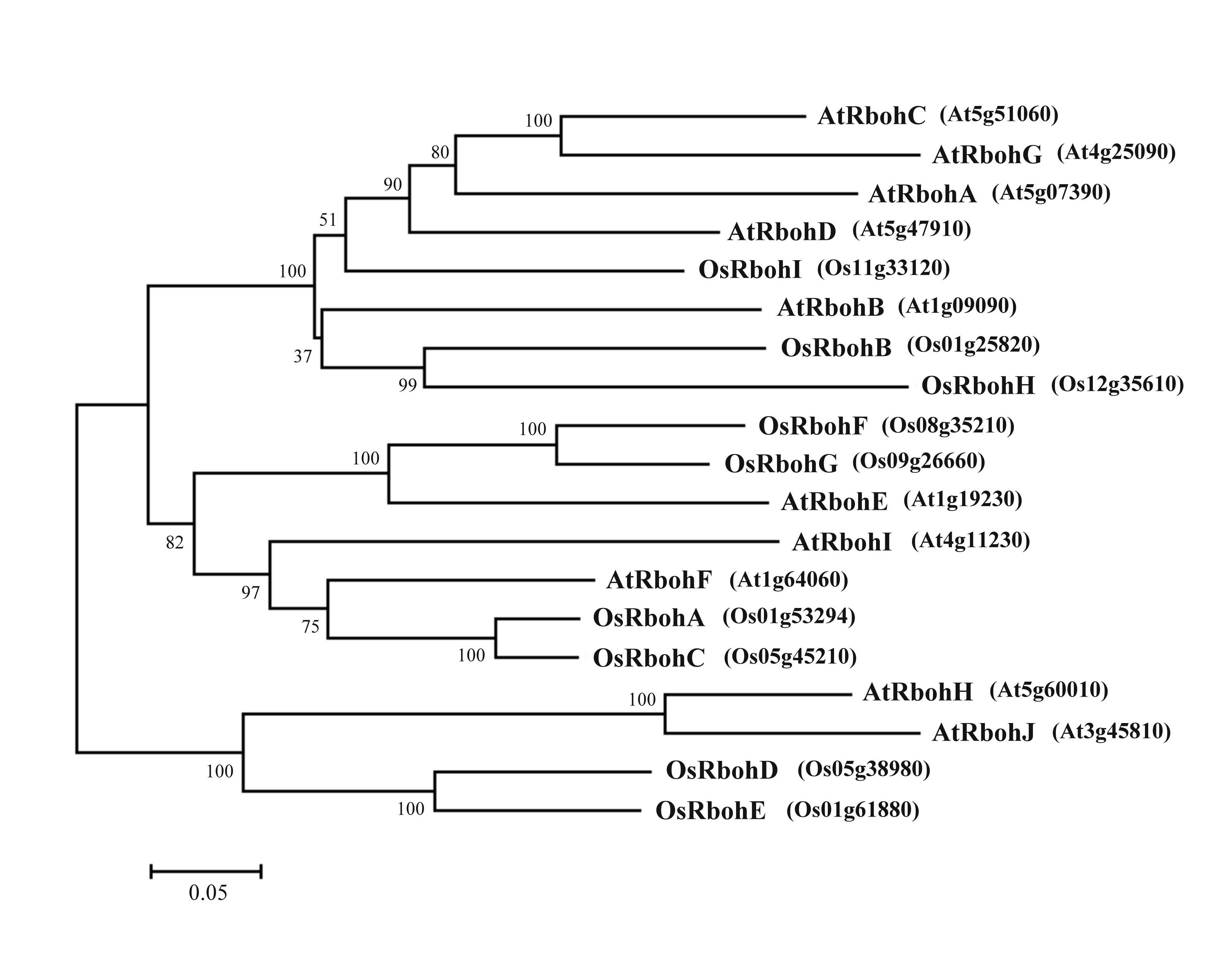

Supplement: Additional file 2: Figure S2. — Phylogenetic tree of members of Rbohs protein family. Phylogenetic tree of 10 Arabidopsis Rboh proteins and 9 rice (Oryza sativa) related Rboh proteins. The phylogenetic tree was constructed by the Neighbor Joining algorithm (Saitou and Nei 1987) implemented in the MEGA 6 software package (Tamura et al. 2013). (JPG 614 kb) [file 12284_2016_74_MOESM2_ESM.jpg]
